# Identification of Eight Spliceogenic Variants in BRCA2 Exon 16 by Minigene Assays

**DOI:** 10.3389/fgene.2018.00188

**Published:** 2018-05-24

**Authors:** Eugenia Fraile-Bethencourt, Alberto Valenzuela-Palomo, Beatriz Díez-Gómez, Alberto Acedo, Eladio A. Velasco

**Affiliations:** ^1^Splicing and Genetic Susceptibility to Cancer, Instituto de Biología y Genética Molecular, Consejo Superior de Investigaciones Científicas, Universidad de Valladolid, Valladolid, Spain; ^2^Biome Makers Inc., San Francisco, CA, United States

**Keywords:** breast cancer, BRCA2, DNA variants, splicing, hybrid minigenes

## Abstract

Genetic testing of *BRCA1* and *BRCA2* identifies a large number of variants of uncertain clinical significance whose functional and clinical interpretations pose a challenge for genetic counseling. Interestingly, a relevant fraction of DNA variants can disrupt the splicing process in cancer susceptibility genes. We have tested more than 200 variants throughout 19 BRCA2 exons mostly by minigene assays, 54% of which displayed aberrant splicing, thus confirming the utility of this assay to check genetic variants in the absence of patient RNA. Our goal was to investigate *BRCA2* exon 16 with a view to characterizing spliceogenic variants recorded at the mutational databases. Seventy-two different BIC and UMD variants were analyzed with NNSplice and Human Splicing Finder, 12 of which were selected because they were predicted to disrupt essential splice motifs: canonical splice sites (ss; eight variants) and exonic/intronic splicing enhancers (four variants). These 12 candidate variants were introduced into the BRCA2 minigene with seven exons (14–20) by site-directed mutagenesis and then transfected into MCF-7 cells. Seven variants (six intronic and one missense) induced complete abnormal splicing patterns: c.7618-2A>T, c.7618-2A>G, c.7618-1G>C, c.7618-1G>A, c.7805G>C, c.7805+1G>A, and c.7805+3A>C, as well as a partial anomalous outcome by c.7802A>G. They generated at least 10 different transcripts: Δ16p_44_ (alternative 3’ss 44-nt downstream; acceptor variants), Δ16 (exon 16-skipping; donor variants), Δ16p_55_ (alternative 3’ss 55-nt downstream), Δ16q_4_ (alternative 5’ss 4-nt upstream), Δ16q_100_ (alternative 5’ss 4-nt upstream), ▾16q^20^ (alternative 5’ss 20-nt downstream), as well as minor (Δ16p_93_ and Δ16,17p_69_) and uncharacterized transcripts of 893 and 954 nucleotides. Isoforms Δ16p_44_, Δ16, Δ16p_55_, Δ16q_4_, Δ16q_100_, and ▾16q^20^ introduced premature termination codons which presumably inactivate BRCA2. According to the guidelines the American College of Medical Genetics and Genomics these eight variants could be classified as pathogenic or likely pathogenic whereas the Evidence-based Network for the Interpretation of Germline Mutant Alleles rules suggested seven class 4 and one class 3 variants. In conclusion, our study highlights the relevance of splicing functional assays by hybrid minigenes for the clinical classification of genetic variations. Hence, we provide new data about spliceogenic variants of BRCA2 exon 16 that are directly correlated with breast cancer susceptibility.

## Introduction

Hereditary Breast and Ovarian Cancer (HBOC) represents 5–10% of all breast cancers. Nowadays, more than 25 HBOC susceptibility genes have been identified, most of them involved in DNA repair pathways (Nielsen et al., 2016). Deleterious variants of the most prevalent genes *BRCA1* (MIM# 113705) and *BRCA2* (MIM# 600185) confer up to 87% of risk to develop breast cancer by the age of 70 years (Petrucelli et al., 2013). Apart from specific founder deleterious mutations (Levy-Lahad et al., 1997; Infante et al., 2013), there have been described thousands of different BRCA1/2 variants at the mutation databases. According to Universal Mutation Database (UMD, http://www.umd.be; date last accessed 2017/06/16) 2,495 and 3,454 different variants have been detected in *BRCA1* and *BRCA2*, respectively, where a relevant fraction of them has been classified as variants of uncertain significance (VUS). These pose a challenge in clinical genetics since mutation carriers could benefit from preventive and prophylactic measures as well as new targeted therapies such as the Poly-ADP Ribose Polymerase Inhibitors (Ricks et al., 2015).

Standard approaches tend to classify DNA variants from the protein point of view. In this way, nonsense variants and frameshift insertions and deletions are automatically classified as pathogenic if they truncate critical protein domains [Evidence-based Network for the Interpretation of Germline Mutant Alleles (ENIGMA) class 5^1^]. However, upstream gene expression mechanisms, such as splicing, can be disrupted by DNA changes. In fact, splicing is a critical highly regulated process involved in many cell functions whose disruption has been directly related with disease, being common in cancer (Wang and Cooper, 2007; Douglas and Wood, 2011). Likewise, spliceogenic variants are more common than they are thought, and they are not restricted to the sequences of the canonical donor and acceptor sites since it has been suggested that up to 50% of exon variants could also affect splicing (López-Bigas et al., 2005). This can be explained by the wide range of splicing regulatory elements (SREs) that control this process, which include the conserved splice sites (5’ss and 3’ss), the branch point, polypyrimidine track, exonic/intronic splicing enhancers (ESEs/ISEs) and exonic/intronic splicing silencers (ESSs/ISSs) (Grodecká et al., 2017), as well as other regulatory components or the RNA secondary structure (Soemedi et al., 2017). Thus, all these factors cooperate with splicing factors and the spliceosome, to accurately remove introns (Will and Lührmann, 2011).

Interestingly, spliceogenic variants are often found in *BRCA2*. Our previous results showed that more than a half of tested *BRCA2* variants impaired splicing (Acedo et al., 2012, 2015; Fraile-Bethencourt et al., 2017). Moreover, the minigene technology was confirmed as a reliable tool to functionally assay potential splicing variants. Here, we aimed to check *BRCA2* exon 16 candidate variants to characterize the splicing effects using the pSAD-based minigene MGBR2_14-20, previously employed to assay DNA variants of exons 17 and 18 (Fraile-Bethencourt et al., 2017). We have assayed 12 likely spliceogenic variants from HBOC patients reported in databases and selected after bioinformatics predictions. Wild-type (wt) and mutant minigenes assays showed that eight variants altered the splicing. Thus, we provide valuable information of spliceogenic *BRCA2* exon 16 variants that could be classified following ENIGMA and American College of Medical Genetics and Genomics (ACMG) guidelines (Richards et al., 2015).

## Materials and Methods

Ethical approval for this study was obtained from the Ethics Review Committee of the Hospital Universitario Río Hortega de Valladolid (6/11/2014).

### Variant Collection and *In Silico* Analyses

*BRCA2* introns 15 and 16 and exon 16 variants were collected from the BIC database^2^ and the BRCA Share Database (UMD, date last accessed 2017/06/16; http://www.umd.be/BRCA2/) (Beroud et al., 2016). Variant descriptions were according to the *BRCA2* GenBank sequence NM000059.1 and the guidelines of the Human Genome Variation Society (HGVS^3^).

Wild-type and mutant sequences were analyzed with NNSPLICE^4^ (Reese et al., 1997) and Human Splicing Finder version 3.0 (HSF^5^) (Desmet et al., 2009), which includes algorithms to detect splice sites, branch point, silencers, and enhancers (Fairbrother et al., 2002; Cartegni et al., 2003; Sironi et al., 2004; Wang et al., 2004; Yeo and Burge, 2004; Zhang and Chasin, 2004).

### Minigene and Mutagenesis

MGBR2_ex14-20 was assembled as previously described (Fraile-Bethencourt et al., 2017). DNA variants and deletions were introduced by the QuikChange Lightning Kit (Agilent, Santa Clara, CA, United States). The wt minigene MGBR2_ex14-20 was used as template to generate 12 BIC/BRCA Share DNA variants and 4 microdeletions (**Table 1**). They were checked by SANGER sequencing at the Macrogen Spain facility (Macrogen, Madrid, Spain).

**Table 1:** Mutagenesis primers of candidate splicing variants.

**HGVS variants**	**Primers (5′ → 3′)**
c.7618-2A>T	GTGTGTTTATTTTGTGTTGCTGTATACGTATGGCG
	CGCCATACGTATACAGCAACACAAAATAAACACAC
c.7618-2A>G	GTGTGTTTATTTTGTGTGGCTGTATACGTATGGCG
	CGCCATACGTATACAGCCACACAAAATAAACACAC
c.7618-1G>A	GTGTGTGTTTATTTTGTGTAACTGTATACGTATGGCGTTTC
	GAAACGCCATACGTATACAGTTACACAAAATAAACACACAC
c.7618-1G>C	GTGTGTGTTTATTTTGTGTACCTGTATACGTATGGCGTTTC
	GAAACGCCATACGTATACAGGTACACAAAATAAACACACAC
c.7625C>G	TTATTTTGTGTAGCTGTATAGGTATGGCGTTTCTAAACATT
	AATGTTTAGAAACGCCATACCTATACAGCTACACAAAATAA
c.7738C>T	ATGGACTGGAAAAGGAATATAGTTGGCTGATGGTGGATGG
	CCATCCACCATCAGCCAACTATATTCCTTTTCCAGTCCAT
c.7753G>A	AATACAGTTGGCTGATGGTAGATGGCTCATACCCTCCAAT
	ATTGGAGGGTATGAGCCATCTACCATCAGCCAACTGTATT
c.7772A>G	GGATGGCTCATACCCTCCAGTGATGGAAAGGCTGGAAAAG
	CTTTTCCAGCCTTTCCATCACTGGAGGGTATGAGCCATCC
c.7802A>G	GCTGGAAAAGAAGAATTTTGTAGGTACTCTATGCAAAAAG
	CTTTTTGCATAGAGTACCTACAAAATTCTTCTTTTCCAGC
c.7805G>C	GGAAAAGAAGAATTTTATACGTACTCTATGCAAAAAGATT
	AATCTTTTTGCATAGAGTACGTATAAAATTCTTCTTTTCC
c.7805+1G>A	GGAAAAGAAGAATTTTATAGATACTCTATGCAAAAAGATTG
	CAATCTTTTTGCATAGAGTATCTATAAAATTCTTCTTTTCC
c.7805+3A>C	AAGAAGAATTTTATAGGTCCTCTATGCAAAAAGATTG
	CAATCTTTTTGCATAGAGGACCTATAAAATTCTTCTT
**Microdeletions**	**Primers (5′ → 3′)**
c.7620_7649del	TTGTGTGTGTTTATTTTGTGTAGCTAAAAATTAACAGCAAA
	AATGCAGAG
	CTCTGCATTTTTGCTGTTAATTTTTAGCTACACAAAATAAAC
	ACACACAA
c.7645_7674del	GTATACGTATGGCGTTTCTAAACATTCTTTTCAGTTTCACAC
	TGAAGATT
	AATCTTCAGTGTGAAACTGAAAAGAATGTTTAGAAACGCCA
	TACGTATAC
c.7748_7772del	ACTGGAAAAGGAATACAGTTGGCTGGAAAGGCTGGAAAAG
	AAGAATTTTA
	TAAAATTCTTCTTTTCCAGCCTTTCCAGCCAACTGTATTCCT
	TTTCCAGT
c.7773_7802del	ATGGTGGATGGCTCATACCCTCCAATAGGTACTCTATGCAA
	AAAGATTGT
	ACAATCTTTTTGCATAGAGTACCTATTGGAGGGTATGAGCC
	ATCCACCAT

### Transfection of Eukaryotic Cells

MCF-7 cells were plated (∼2 × 10^5^ cells/well) and grown to 90% confluency in 0.5 mL of medium (MEME, 10% fetal bovine serum, 2 mM glutamine, 1% non-essential amino acids, and 1% penicillin/streptomycin) in four-well plates (Nunc, Roskilde, Denmark). Transfections were made with 1 μg of minigene and 2 μL of low toxicity Lipofectamine (Life Technologies, Carlsbad, CA, United States) in Gibco^TM^ Opti-Mem^TM^ (Thermo Fisher Scientific, Waltham, MA, United States). Cells were incubated with 300 μg/mL of cycloheximide (Sigma-Aldrich, St. Louis, MO, United States) for 4 h to inhibit nonsense-mediated decay (NMD). RNA was purified with the Genematrix Universal RNA Purification Kit (EURx, Gdansk, Poland) with on-column DNAse I digestion to degrade genomic DNA that could interfere with RT-PCR.

### RT-PCR of Minigenes

Approximately 400 ng of RNA was retrotranscribed using RevertAid H Minus First Strand cDNA Synthesis Kit (Life Technologies, Carlsbad, CA, United States) and the gene-specific primer RTPSPL3-RV (5′-TGAGGAGTGAATTGGTCGAA-3′). Samples were incubated at 42°C for 1 h, and reactions were inactivated at 70°C for 5 min. Then, 40 ng of cDNA was amplified in 50 μL reaction with pMAD_607FW (Patent P201231427, CSIC) and RTBR2_ex17RV2 (5′-GGCTTAGGCATCTATTAGCA-3′) or with RT_ex15FW (5′-CGAATTAAGAAGAAACAAAGG-3′) and pSAD_RT_RV (Patent P201231427, CSIC) using Platinum Taq DNA polymerase (Life Technologies, Carlsbad, CA, United States) (size of transcripts: 1018 and 1250 nt, respectively). Samples were denatured at 94°C for 2 min, followed by 35 cycles consisting of 94°C for 30 s, Td-2°C for 30 s, and 72°C (1 min/kb), and a final extension step at 72°C for 5 min. Sequencing reactions were performed by the sequencing facility of Macrogen Spain. Semi-quantitative fluorescent 26 cycles PCRs were done in triplicate with primers pMAD_607FW-FAM and RTBR2_ex17RV2 using Platinum Taq DNA polymerase (Life Technologies, Carlsbad, CA, United States). FAM-labeled products were run with Genescan LIZ-1200 as size standard (Life Technologies, Carlsbad, CA, United States) at the Macrogen facility and analyzed with the Peak Scanner software V1.0. Only peaks with heights ≥50 relative fluorescence unit (RFU) were considered. Mean peak areas of each transcript of three runs were used to quantify the relative abundance of each transcript.

## Results

### Bioinformatics Analysis of Splicing Variants

Seventy-two variants were collected from the BIC and UMD databases. Among them, 35 had been previously classified as VUS and 34 as pathogenic or likely pathogenic. In order to select possible spliceogenic variants, they were analyzed by the splicing prediction software NNSplice and HSF (Supplementary Table S1). Selections were made following the next criteria: (a) ss creation or disruption; (b) branch point disruption; and (c) ESS creation (hnRNPA1). Curiously, NNSplice did not recognize exon 16 canonical 5′ss. In contrast, a very strong 100-nt upstream cryptic donor (NNSplice score: 0.99) was identified at position c.7706_7707. The MaxEnt results showed a weak canonical donor (4.68) and a strong cryptic donor (8.92) (**Table 2**).

**Table 2:** Bioinformatics analysis of potential splicing variants of *BRCA2* exon 16.

DNA variants	NNSplice^1^	MaxEnt^1^	ESE finder^2^	ESE HSF^2^	hnRNPA1^2^
Ex16-Canonical ss	3′SS: 0.69	3′SS: 7.11			
	5′SS <0.4	5′SS: 4.68			
c.7618-2A>T	[-] 3′SS: 0.69 → <0.4	[-] 3′SS: 7.11 →-1.24			[-] (70.24)
c.7618-2A>G	[-] 3′SS: 0.69 → <0.4	[-] 3′SS: 7.11 →-0.83	[-] SRp55 (83.78)		[-] (70.24)
c.7618-1G>A	[-] 3′SS: 0.69 → <0.4	[-] 3′SS: 7.11 →-1.63	[-] SRp55 (77.06)		[-] (70.24)
c.7618-1G>C	[-] 3′SS: 0.69 → <0.4	[-] 3′SS: 7.11 →-0.94	[-] SRp55 (77.06)		[-] (70.24)
c.7625C>G (p.Thr2542Arg)	[+] 3′SS (<0.4 → 0.94); [+]	[+] 5′SS (2.23 → 7.37)	[-] SRp55(89.80)	[-] 9G8 (62.28)	[+] (88.33)
	5′SS (<0.4 → 0.91)				
c.7738C>T (p.Gln2580Ter)			[-] SC35 (81.01)		[+] (70.24)
c.7753G>A (p.Gly2585Arg)		[+] 5′SS (-1.33 → 3.06)			[+] (74.05)
c.7772A>G (p.Asn2591Ser)			[+] SF2/ASF (IgM) (74.85)	[-] 9G8 (60.94)	[+] (69.52)
c.7802A>G (p.Tyr2601Cys)			[-] SRp40 (83.89)		[-] (66.43)
c.7805G>C (p.Arg2602Thr)		[-] 5′SS: 4.68 →-2.79	[+] SRp55 (89.80)		[-] (69.05)
c.7805+1G>A		[-] 5′SS: 4.68 →-3.50	[-] SRp40 (83.89);		
			[-] SC35 (82.36)		
c.7805+3A>C		[-] 5′SS: 4.68 →-3.85			

*[+] and [-] symbols indicate creation or disruption of splicing motifs, respectively. Thresholds of the splicing programs: ^1^Splice sites (ss): NNSPLICE (values 0–1): Cut-offs = 0.4 for both 5′- and 3′ ss (ss disruption <0.4, ss creation >0.4); MaxEnt: 3.0 for 5′ and 3′ ss (variation threshold ± 30% according to HSF). ^2^Enhancers and silencers HSF: Human Splicing Finder matrices (default values, http://www.umd.be/HSF3/technicaltips.html): ESEfinder cut-offs (HSF scale, normalized to 0–100): SF2/ASF: 72.98/SF2/ASF (IgM-BRCA1): 70.51/SRp40: 78.08/SC35: 75.05/SRp55: 73.86; ESE motifs from HSF, cut-offs values (0–100): Tra2: 75.964/9G8: 59.245; hnRNP motifs: hnRNPA1: 65.476 (these values were considered the limits for the disruption or creation of a splicing regulatory element)*.

**FIGURE 1:**
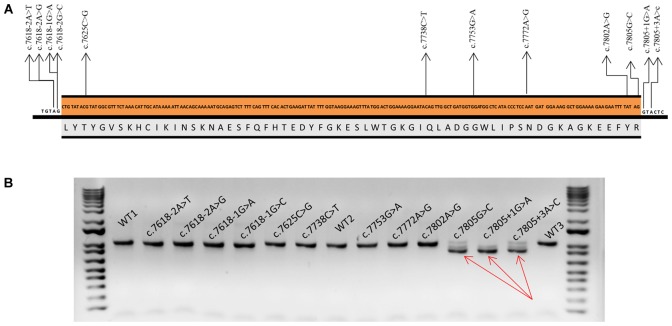
Functional assays of spliceogenic candidate variants of *BRCA2* exon 16. **(A)** Nucleotide (c.7618_7805) and amino acid (p.2540_2602) sequences of *BRCA2* exon 16. Arrows indicate selected variants. **(B)** Agarose gel electrophoresis of RT-PCR products of the wt and mutant minigenes and the size standard 1 Kb Plus DNA Ladder at both sides of the gel. Amplification was made with primers pMAD607-FW and RTBR2_ex17RV2. Full-length transcript (V1-EX17) size: 1018 nt. Red arrows point to exon 16 skipping band (Δ16) (size: 830 nt).

Twelve variants were selected (**Figure 1**): six intronic (c.7618-2A>T, c.7618-2A>G, c.7618-1G>C, c.7618-1G>A, c.7805+1G>A, and c.7805+3A>C), five missense (c.7625C>G, c.7753G>A, c.7772A>G, c.7802A>G, and c.7805G>C), and one nonsense (c.7738C>T) variants. Four missense variants (c.7625C>G, c.7753G>A, c.7772A>G, and c.7802A>G) and c.7805+3A>C had been previously classified as VUS. Intronic variants (c.7618-2, -1 and c.7805+1, +3) and c.7805G > C disrupted the canonical ss, whereas variants c.7625C>G and c.7753G>A created new ss. DNA change c.7802A>G was selected because of its proximity to the canonical donor site and the presumable generation of an alternative “gt” donor site 4-nt upstream (underlined, TTTGTAGgtactc). Finally, bioinformatics results of c.7738C>T and c.7772A>G suggested the creation of one ESS (hnRNPA1) (**Table 2**).

### Splicing Functional Assays of DNA Variants

The minigene MGBR2_ex14-20 had been already shown as a robust tool to assay possible spliceogenic variants contained in any of those exons and flanking introns (Fraile-Bethencourt et al., 2017). The wt construct produced a full-length transcript of the expected size (1806 nt), sequence, and structure (V1-BRCA2 exons 14-20-V2). To map the presence of putative splicing enhancers, a set of four overlapping exonic microdeletions were generated, which spanned 55-nt of the 5′- and 3′-ends (Fairbrother et al., 2004). This strategy had been previously shown to increase the accuracy of predictions of ESE disrupting variants (Acedo et al., 2015; Fraile-Bethencourt et al., 2017). None of the microdeletions induced splicing anomalies suggesting that this exon is not controlled by ESEs (data not shown). Consequently, ESE-disrupting variants, as unique selection criterion, were not chosen for subsequent functional tests (**Table 2**).

**FIGURE 2:**
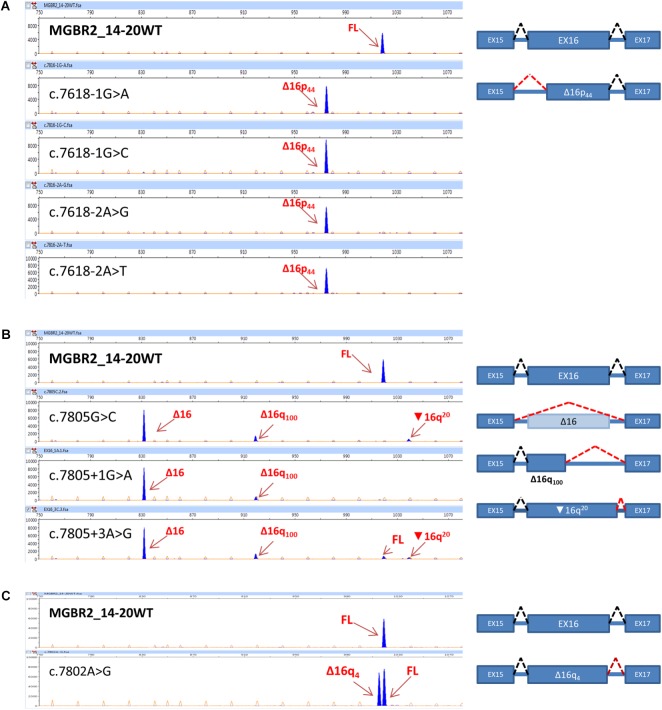
Fluorescent capillary electrophoresis of transcripts from *BRCA2* exon 16 variants. On the left, screenshots of electropherograms are shown. cDNA was amplified with primers FAM-labeled pMAD_607FW and RTBR2_ex17RV2. Arrows indicate transcripts (blue peaks). Full-length transcript: 1018 nt. Size standard was Genescan LIZ 1200 (orange/faint peaks). Fragments were analyzed with the Peak Scanner software v1.0. Fragment sizes (bp) and relative fluorescent units are indicated on the *x*- and *y*-axes, respectively. On the right, diagrams of the splicing patterns are shown. Boxes represent exons, discontinued black lines represent canonical splicing, and discontinue red lines represent aberrant splicing. **(A)** Acceptor site variants. **(B)** Donor site variants. **(C)** Alternative donor variant.

Selected variants were introduced into the minigene and functionally assayed in MCF-7 cells. Agarose electrophoresis clearly showed that three of them (c.7805G>C, c.7805+1G>A, and c.7805+3A>C) induced aberrant splicing patterns (**Figure 1**). However, the high resolution and sensitivity of fluorescent capillary electrophoresis allowed us to identify a total of eight variants, including the three previous ones, that disrupted splicing: c.7618-2A>T, c.7618-2A>G, c.7618-1G>C, c.7618-1G>A, c.7802A>G, c.7805G>C, c.7805+1G>A, and c.7805+3A>C (**Figure 2**). Actually, this approach is able to detect rare transcripts with a relative abundance below 1% or can resolve transcripts that differ only in a few nucleotides (e.g., only 4-nt between the canonical and Δ16q4 isoforms). A total of at least 10 different aberrant transcripts were characterized by fragment analysis and sequencing: Δ16p44 (44-nt deletion; alternate 3′ss 44-nt downstream), Δ16p55 (55-nt del; alternate 3′ss 55-nt downstream), Δ16 (exon 16 skipping), Δ16q4 (4-nt del; alternate 5′ss 4-nt upstream), Δ16q100 (100-nt del; alternate 5′ss 100-nt upstream), ▾16q20 (20-nt insertion; alternate 5′ss 20-nt downstream), minor (Δ16p_93_ and Δ16,17p_69_), and uncharacterized transcripts of 893 and 954 nt (**Figure 2** and **Table 3**). On the one hand, fragments analysis and sequences revealed that 3′ss disrupting variants (positions -2 and -1) provoked the use of a cryptic acceptor 44-nt downstream (Δ16p_44_) within exon 16 (**Figure 2A**). Interestingly, this cryptic 3′ss was not recognized either by NNSplice or MaxtEnt. The loss of 44-nt at 5′ of exon 16 would suppose a frameshift deletion and a premature termination codon (PTC) (p.L2540Qfs^∗^11). On the other hand, 5′ss variants (positions +1 and +3) produced exon 16 skipping (Δ16), which means a frameshift deletion through the loss of 188-nt from r.7618 to r.7805 (**Figure 2B**). Consequently, BRCA2 would be truncated with a PTC four codons downstream (p.L2540Gfs^∗^4). Last exon nucleotide variant (c.7805G>C) induced the same outcome (Δ16) highlighting the importance of this position conservation (G in nearly 80% in all exons) in exon recognition (Zhang, 1998). Fragment analysis of variants c.7805G>C, c.7805+1G>A, and c.7805+3A>C also showed ∼14% of transcript Δ16q_100_, which corresponds with the use of the previously mentioned cryptic 5′ss within exon 16 (NNSplice: 0.99; MaxEnt: 8.92), provoking r.7706_7805del (p.K2570Lfs^∗^45) (**Table 3** and **Figure 2B**). Finally, missense variant c.7802A>G created a new 5′ss, which resulted in ∼45% of the aberrant transcript Δ16q_4_ (**Figure 2C**). The loss of four nucleotides would introduce a PTC into the protein (p.Y2601Wfs^∗^46). Thus, our results showed clearly how these eight variants disrupted splicing. Moreover, seven of them (c.7618-2A>T; c.7618-2A>G; c.7618-1G>C; c.7618-1G>A; c.7805G>C; c.7805+1G>A; c.7805+3A>C) generated more than ∼92% of frameshift transcripts.

**Table 3:** Quantification of transcripts of spliceogenic variants of exon 16 by fluorescent capillary electrophoresis.

	Canonical	Δ16p_44_	Δ16p_55_	Δ16	Δ16q_4_	Δ16q_100_	▾16q^20^	Others
MGBR2_14-20	100%							
DNA variants								
c.7618-2A>T		96.9 ± 0.4%	1.8 ± 0.3%	1.3 ± 0.03%				
c.7618-2A>G		97.2 ± 0.4%						2.8 ± 0.4% (954 nt)
c.7618-1G>A		91.5 ± 0.2%	4.7 ± 0.2%	0.7 ± 0.03%				1.7 ± 0.03% (Δ16p_93_);
								1.4 ± 0.04% (Δ16,17p_69_)
c.7618-1G>C		92.6 ± 0.3%	1.9 ± 0.2%	2.8 ± 0.05%				1.1 ± 0.05% (Δ16p_93_)
								0.7 ± 0.03%0.7 ± 0.03% (893 nt)
								0.9 ± 0.07% (Δ16,17p_69_)
c.7625C>G	100%							
c.7738C>T	100%							
c.7753G>A	100%							
c.7772A>G	100%							
c.7802A>G	54.3 ± 0.3%				45.7 ± 0.3%			
c.7805G>C				77.6 ± 0.6%		14.4 ± 0.3%	6.5 ± 0.3%	1.5 ± 0.06% (Δ16,17p_69_)
c.7805+1G>A				88.0 ± 0.2%		10.1 ± 0.1%		1.9 ± 0.02% (Δ16,17p_69_)
c.7805+3A>C	7.6 ± 0.2%			75.3 ± 0.5%		13.3 ± 0.2%	3.8 ± 0.3%	

*HGVS-RNA effect of transcripts: **Δ**16p_44_, r.7618_7661del; Δ16q_4_, r.7802_7805del; Δ16, r.7618_7805del; Δ16q_100_, r.7706_7805del; Δ16p_55_, r.7618_7672del; ▾16q^20^, r.7805_7806ins7805+1_7805+20; Δ16p_93_, r.7618_7710del; Δ16,17p_69_, r.7618_7874del*.

## Discussion

Nowadays, with the advent of new generation sequencing technologies and, namely, cancer-gene panels (Slavin et al., 2015), thousands of variants are being described. However, their classifications as neutral or deleterious variants pose a challenge in Human Genetics. In fact, some deleterious variants can be missed because they are synonymous or intronic. Moreover, a significant fraction of *BRCA2* variants are considered VUS and require additional proofs to be reclassified, including functional tests. Here, we have shown that the minigene MGBR2_14-20 is a robust tool to functionally assay candidate spliceogenic variants of the *BRCA2* exon 16. Until now, we have comprehensively studied candidate splicing variants from 20 out of 27 *BRCA2* exons (Sanz et al., 2010; Acedo et al., 2012, 2015; Fraile-Bethencourt et al., 2017). Thus, we have found six intronic and two missense *BRCA2* variants which alter the splicing and could confer cancer risk.

*BRCA2* exon 16 codifies from Leucine 2540 to Arginine 2602 (p.2540_2602). Interestingly, according to the International Agency for Research on Cancer (IARC^6^), this is a conserved region, since there is ∼22% of ultra-conserved aminoacids from human to sea urchin and ∼54% between mammals. Furthermore, this protein segment belongs to FANCD2- and DSS1-binding domains. Fanconi Anemia group D2 (FANCD2) protein binds to aminoacids from position p.2350 to p.2545 of BRCA2 and it has been suggested to have a role in the repair process (Hussain et al., 2004). DSS1 (Delete in Split hand/Split foot) protein, which binds to BRCA2 at positions p.2467_2957 (Marston et al., 1999), is an essential element of BRCA2 stability, since its loss supposes a dramatic decrease of BRCA2 levels (Li et al., 2006). Altogether, this highlights the value of exon 16 in BRCA2 function. Moreover, exon 16 skipping supposes a frame-shift deletion and the generation of a PTC (p.L2540Gfs^∗^4), which would truncate the protein and subsequently loss the C-terminal region that would compromise BRCA2 function.

This study, based on minigene technology, provides detailed information about the impact on splicing of 12 *BRCA2* exon 16 variants. Aberrant splicing outcomes were found in eight of these variants, six intronic and two missense changes. Intriguingly, none of the aberrant transcripts described here was previously reported as natural alternative splicing events of the *BRCA2* gene (Fackenthal et al., 2016). Among them, seven (c.7618-2A>T, c.7618-2A>G, c.7618-1G>A, c.7618-1G>C, c.7805G>C, c.7805+1G>A, and c.7805+3A>C) provoked more than ∼92% of frameshift transcripts. Interestingly, previous studies of variant c.7618-1G>A in lymphoblastoid cells showed that 3′ss disruption induced transcripts Δ16p_44_ and Δ16,17p_69_ (Whiley et al., 2011). Here we found both transcripts, but also other minor ones: Δ16p_55_, Δ16p_93_, Δ16 (**Table 3**). Additionally, according to our data Δ16p_44_ is the main transcript (∼91%) that other authors also identified but described as a minor transcript in agarose gels (Whiley et al., 2011). These differences could be due to: (i) the cell line; (ii) the use of cycloheximide to inhibit the NMD; (iii) the fact that we work with a single-mutant allele, avoiding the wt counterpart effect; and (iv) the high sensitivity of fluorescent capillary electrophoresis, which can detect rare transcripts versus agarose electrophoresis. In any case, both results show that c.7618-1G>A severely disrupted splicing. On the other hand, variant c.7805G>C was previously reported to result in Δ16 and Δ16q_100_, with the total absence of the canonical transcript (Bonnet et al., 2008). This outcome matches our results (**Table 3**): Δ16 as the main transcript (∼78%), followed by Δ16q_100_ (∼14%), and the lack of the full-length transcript. It is also worthy to mention that we detected other minor transcripts due to the high sensitivity of fluorescent capillary electrophoresis (▾16q^20^ at ∼6.5% and Δ16,17p_69_ at ∼1.5%) that otherwise could not be easily detected on agarose gels. In any case, the spliceogenic effects of variants c.7618-1G>A and c.7805G>C were supported by our data.

Variant c.7802A>G probably generated the most conflicting result since it triggered ∼54% of canonical transcript and ∼46% of Δ16q_4_, so that its interpretation is more complex. The transcript Δ16q_4_, caused by the use of a new 5′ss, generated a frameshift deletion and the protein truncation by a PTC 46 codons downstream (p.Y2601Wfs^∗^46). However, it is still unclear if ∼54% of full-length transcript can preserve BRCA2 function, given that, for example, 20–30% of *BRCA1* transcript is able to maintain BRCA1 activity (de la Hoya et al., 2016). It is also important to keep in mind that full-length transcript carries a missense variant (p.Y2601W) that, according to IARC alignment^7^, Tyrosine 2601 is highly conserved from human to sea urchin, suggesting an important function in the protein. Moreover, PolyPhen-2 (Adzhubei et al., 2010) predicted that this aminoacid change is damaging with the maximum score (1.0). Curiously, c.7802A>G was reported a family with a significant history of primary cancers (colorectal, lymphoma, and breast cancers) which carried biallelic *BRCA2* mutations (c.7802A>G and c.1845_1856delCT). However, patients did not present the typical FA phenotype, which suggested that p.Y2601W BRCA2 maintained at least enough BRCA2 activity to prevent early childhood FA features (Degrolard-Courcet et al., 2014). Nevertheless, this missense change remains classified as VUS in ClinVar^8^.

On the other hand, variant c.7625C>G was previously computed to disrupt one SRp55 motif (Pettigrew et al., 2008), although functional mapping by microdeletions indicated that exon 16 is likely not regulated by splicing enhancers. Nevertheless, this change was selected because it presumably created new strong 3′ and 5′ss as well, both with a NNSplice score >0.9 (**Table 2**). However, c.7625C>G only produced the full-length transcript without any splicing anomaly. The protein would even carry the missense variant p.T2542R. However, consistent with PolyPhen, this change might be considered as benign with a score of 0.0, which could be explained by the low conservation of the affected threonine. Anyway, further functional and association studies must be performed to interpret this variant. Other variant that resulted in a normal splicing pattern was the nonsense variant c.7738C>T (p.Q2580X), that *a priori* had been classified as pathogenic. In this case, the protein would be truncated at codon 2580 losing 839 aminoacids of the C-terminal where the DSS1-binding site, the DNA-binding domain, the RAD51C-binding site, and the cyclin-dependent kinase (CDK) phosphorylation site are located (Roy et al., 2012). Interestingly, this variant was found in an Italian non-Ashkenazi *BRCA1* and *BRCA2* double heterozygote family (Musolino et al., 2005).

According to the ACMG guidelines (**Table 4**; Richards et al., 2015), five variants (c.7618-2A>T, c.7618-2A>G, c.7618-1G>A, c.7618-1G>C, and c.7805+1G>A) can be classified as pathogenic as they match criteria PVS1 (very strong evidence of pathogenicity: null variant – nonsense, frameshift, canonical ±1 or 2 ss, initiation codon, single or multiexon deletion – in a gene where LOF is a known mechanism of disease), PS3 (strong evidence: well-established *in vitro* or *in vivo* functional studies supportive of a damaging effect on the gene or gene product), PM2 (moderate evidence: absent from controls in Exome Sequencing Project, 1000 Genomes Project, or Exome Aggregation Consortium), PP3 (supporting evidence: multiple lines of computational evidence support a deleterious effect on the gene or gene product: conservation, evolutionary, splicing impact, etc.), and PP5 (reputable source recently reports variant as pathogenic, but the evidence is not available to the laboratory to perform an independent evaluation). On the other hand, variants c.7802A>G, c.7805G>C, and c.7805+3A>C were classified as likely pathogenic as they match criteria PS3, PM2, PP3, and PP5.

**Table 4:** Classification of variants according to the ENIGMA and ACMG rules.

DNA variants	Main aberrant transcripts^1^	HGVS RNA/protein effects	Previous classification^2^	ACMG classification^3^	Enigma classification^4^
c.7618-2A>T	Δ16p_44_	r.7618_7661del/p.L2540Qfs^∗^11	5 – Causal	Pathogenic: PVS1, PS3, PM2, PP3, PP5	Class 4
c.7618-2A>G	Δ16p_44_	r.7618_7661del/p.L2540Qfs^∗^11	5 – Causal	Pathogenic: PVS1, PS3, PM2, PP3, PP5	Class 4
c.7618-1G>A	Δ16p_44_	r.7618_7661del/p.L2540Qfs^∗^11	5 – Causal	Pathogenic: PVS1, PS3, PM2, PP3, PP5	Class 4
c.7618-1G>C	Δ16p_44_	r.7618_7661del/p.L2540Qfs^∗^11	5 – Causal	Pathogenic: PVS1, PS3, PM2, PP3, PP5	Class 4
c.7802A>G	Δ16q_4_	r.7802_7805del/p.Y2601Wfs^∗^46	3 – VUS	Likely pathogenic: PS3, PM2, PP3, PP5	Class 3
c.7805G>C	Δ16, Δ16q_100_	r.[7618_7805del; 7706_7805del]/p.[L2540Gfs^∗^4; K2570Lfs^∗^45]	5 – Causal	Likely pathogenic: PS3, PM2, PP3, PP5	Class 4
c.7805+1G>A	Δ16, Δ16q_100_	r.[7618_7805del; 7706_7805del]/p.[L2540Gfs^∗^4; K2570Lfs^∗^45]	5 – Causal	Pathogenic: PVS1, PS3, PM2, PP3, PP5	Class 4
c.7805+3A>C	Δ16, Δ16q_100_	r.[7618_7805del; 7706_7805del]/p.[L2540Gfs^∗^4; K2570Lfs^∗^45]	3 – VUS	Likely pathogenic: PS3, PM2, PP3, PP5	Class 4

*^1^Transcripts were annotated according to previous reports of the ENIGMA consortium (Colombo et al., 2014). Δ, skipping or deletion;* ▾, *insertion; p: alternative acceptor site; q, alternative donor site; subscript number, number of deleted nt; superscript number, number of inserted nt. Thus, Δ16 indicates exon 16 skipping and Δ16p_44_ indicates loss of 44 nt at the exon 16 acceptor site (or new acceptor site 44 nt upstream). All transcripts and their quantification data are described in **Table 2**. ^2^Previous classifications according to the BRCA share (c.7618-2A>T, c.7618-2A>G, c.7618-1G>A, c.7618-1G>C, c.7802A>G, and c.7805G>C) and the BIC mutation databases (c.7805+1G>A and c.7805+3A>C). ^3^ACMG criteria: PVS1, null variant (nonsense, frameshift, canonical ± 1 or 2 ss, etc.) in a gene where LOF is a known mechanism of disease; PS3, well-established in vitro or in vivo functional studies supportive of a damaging effect on the gene or gene product; PM2, absent from controls in Exome Sequencing Project, 1000 Genomes Project, or Exome Aggregation Consortium; PP3, multiple lines of computational evidence support a deleterious effect on the gene or gene product (conservation, evolutionary, splicing impact, etc.); PP5, reputable source recently reports variant as pathogenic, but the evidence is not available to the laboratory to perform an independent evaluation. ^4^ENIGMA. Class 4: Nucleotide positions that are considered extremely likely to alter splicing and/or variants are predicted bioinformatically to alter the use of the native donor/acceptor site. Class 3: “In the absence of clinical evidence to assign an alternative classification, variant allele tested for mRNA aberrations … is found to produce mRNA transcript(s) predicted to encode intact full-length protein ….”*

Similarly, following the ENIGMA rules for variant classification^9^, all variants, except for c.7802A>G, should be reclassified as class 4 (likely pathogenic) because they are “considered extremely likely to alter splicing based on position” and are “predicted bioinformatically to alter the use of the native donor/acceptor site.” Conversely, minigenes are not considered robust approaches to functionally test these variants yet (“… results from construct-based mRNA assays alone are not considered sufficiently robust to be used as evidence for variant classification …”). However, this specific minigene with *BRCA2* exons 14–20 was confirmed as a robust tool since it reproduced patient RNA results from eight variants (Fraile-Bethencourt et al., 2017), and also c.7618-1G>A and c.7805G>C of this study, so that these seven class 4 variants could be even reclassified as class 5. Finally, c.7802A>G was classified as class 3 because it did not meet the above standards and induce a partial aberrant outcome with more than 50% of the canonical transcript.

In summary, we detected eight spliceogenic *BRCA2* exon 16 variants that should be classified as pathogenic or likely pathogenic according to the ACMG guidelines (**Table 4**). Moreover, they account for 22% of causal variants of exon 16 and 11% of all recorded variants of this exon at the mutation databases. Taken together this and our previous studies, we have tested 283 *BRCA1/2* variants under the splicing perspective, 154 of which induced anomalous patterns and 111 could be classified as pathogenic or likely pathogenic. These data remark the importance of variants of splicing regulatory sequences, which are often underestimated because most of them are placed in non-coding regions of the protein. Until now, genetic family-based studies have set up the impact of some variants on cancer risk. However, because of the exponential increment in the number of variants, their low frequencies and different nature, functional assays are strictly required. In this context, minigene technology constitutes a robust tool which can be used to functionally test spliceogenic candidate variants of any disease-gene without the interference of the counterpart wt allele. Certainly, pSAD-based minigenes represented valuable tools to functionally check variants of the SERPINA1 (severe alpha-1 antitrypsin deficiency) and CHD7 (Charge Syndrome) genes (Lara et al., 2014; Villate et al., 2018). RNA assays provide essential data for the initial characterization of VUS and improve the genetic counseling of hereditary diseases.

## Author Contributions

EF-B contributed to the bioinformatics analysis, minigene construction, manuscript writing, and performed most of the splicing functional assays. BD-G and AV-P participated in minigene construction, mutagenesis experiments, and functional assays. AA participated in minigene construction and functional mapping experiments. EV conceived the study and the experimental design, supervised all the experiments, and wrote the manuscript. All authors contributed to data interpretation, revisions of the manuscript, and approved the final version of the manuscript.

## Conflict of Interest Statement

The authors declare that theresearch was conducted in the absence of any commercial or financial relationships that could be construed as a potential conflict of interest.

## Footnotes

1 https://enigmaconsortium.org/library/general-documents/enigma-classification-criteria/

2 https://research.nhgri.nih.gov/projects/bic/Member/index.shtml

3 http://www.hgvs.org/mutnomen/

4 http://www.fruitfly.org/seq_tools/splice.html

5 http://www.umd.be/HSF3/

6 http://agvgd.hci.utah.edu/BRCA2_Spur.html

7 http://agvgd.hci.utah.edu/BRCA2_Spur.html

8 https://www.ncbi.nlm.nih.gov/clinvar/variation/185651/#clinical-assertions

9 https://enigmaconsortium.org/library/general-documents/enigma-classification-criteria/
